# Differential item functioning in the children autism rating scale first edition in children with autism spectrum disorder based on a machine learning approach

**DOI:** 10.3389/fneur.2025.1648991

**Published:** 2025-09-12

**Authors:** Meng Chen, Kanglong Peng, Libing Zhou, Xiaofang Weng

**Affiliations:** ^1^Women and Children’s Health Care Hospital of Luohu, Shenzhen, China; ^2^Shenzhen Children's Hospital, Shenzhen, China

**Keywords:** CARS1, Rasch model, category function, differential item functioning, ASD, machine learning

## Abstract

**Purpose:**

Our study used Rasch Analysis to examine the psychometric properties of the Children Autism Rating Scale First Edition (CARS1) in children with autism spectrum disorder (ASD).

**Methods:**

The Partial Credit Model (PCM) was used to test reliability and validity. The GPCMlasso Model was used to test the differential item functioning (DIF).

**Results:**

The response pattern of this sample showed acceptable fitness for the PCM. This analysis supports the unidimensionality assumption of the CARS1. Disordered category functions and DIF were found for all items in CARS1. Performance can be related to age group, gender, symptom classification, and autistic symptoms.

**Conclusion:**

Rasch analysis provides reliable evidence to support the clinical application of the CARS1. Some items may produce inaccurate measurements originating from unreasonable category structures. Differences in age group, sex, and symptom classification can be related to test performance and may lead to unnecessary bias. Hence, clinical applications may require additional consideration of population characteristics to draw reliable conclusions.

## Introduction

Autism spectrum disorder (ASD) is a highly heritable and heterogeneous neurodevelopmental disorder whose symptoms emerge in the early developmental stage and persist throughout life (1). Currently, the specific pathogenesis mechanism underlying ASD is unknown; hence, no comprehensive cure for ASD has been found (2). Timely diagnosis is required to initiate early interventions, which can lead to more optimal developmental outcomes in individuals with definitive or suspected ASD (3).

A comprehensive ASD diagnosis is established based on a detailed developmental trajectory, clinical decision, and application of standardized diagnostic instruments (3). As no objective evidence can determine whether autistic symptoms fulfil the criteria for ASD diagnosis, the diagnostic decision is mainly based on the clinician’s experience or patients’ self-perception (1, 4).

To promote diagnostic reliability and validity, clinicians tend to describe autistic symptoms in two dimensions according to the Diagnostic and Statistical Manual of Mental Disorders 5th Text Revision (DSM-5-TR): social communication and restricted and repetitive behavior (5). Studies found that the autistic symptoms can be quantitatively rated on differently dimensions underneath the latent structure built based on diagnostic criteria (1, 6–8). Hence, individuals with ASD may present various extreme autistic symptoms in different categories, and clinicians need to decide whether the autistic symptoms are merely autistic-like personalities or true autistic symptoms (9). In addition, researchers have found that individual autistic profiles built based on the DSM-5-TR can be continuously categorized into various subgroups (4). For example, social communication deficits are more common in younger individuals with ASD who present with lower developmental functioning (10). In contrast, those who are older and have higher developmental functioning tend to present restricted and repetitive behaviors (10). This means that the overlap among autistic profiles can be well described by current diagnostic tools, but the variability across different subsamples may jeopardize reliability and validity (11). As reported in previous studies, symptom diversity may originate from the developmental profiles of participants, including age, cognition, speech, and language (4, 12). Hence, special consideration is needed when choosing appropriate diagnostic tools to avoid potential bias caused by latent factors (12).

The Childhood Autism Rating Scale First Edition (CARS1) was developed to depict the symptoms of individuals with ASD and continuously categorize the severity according to the observed functions (13). Since the first case diagnosis as ASD is reported in 1984, the CARS1 is then introduced in China and translated into Chinese in 1988 (14). Accumulating evidence indicates that the CARS1 can be used in clinical settings to describe ASD symptomatology with reasonable psychometric properties (15). Psychometric studies have revealed variability in measurement properties among different subsamples (16). For example, lower inter-rater reliability has been reported in teenagers (0.79), adolescents (0.73), and adults (0.73) (16, 17). In contrast, higher concurrent validity compared to the Autism Diagnostic Interview (ADI) and the Autism Diagnostic Observation Schedule (ADOS) was found in older individuals (17). One cross-cultural study revealed that CARS1 cannot accurately depict autistic symptom in individuals with ASD from different countries and regions (15). To validate the measurement construct, research was conducted to explore the factor structure of CARS (16). The results revealed three dominant dimensions under CARS1, including social communication, stereotyped behaviors and sensory sensitivities, and emotional reactivity; however, the whole testing structure only explained 51.45% of the variance in CARS1 scores (16). More carefully designed psychometric studies are needed to investigate the theoretical structure of the CARS1 to draw more definitive and clinically useful conclusions (18).

In clinical practice, timely diagnosis is critical for individuals with ASD to obtain necessary interventions. Hence, rigorous diagnostic tools are needed to collect reliable messages from individuals who are suspected of suffering from ASD. To scientifically reveal the fundamental structure of CARS1, this study used a probabilistic hypothesis based on Rasch Model to test the item-level psychometric properties in detail. In addition, this study also tries to construct a possible model to elaborate the potential bias brought by demographic variables in clinical settings, and a machine learning approach is utilized to obtain the optimal parameters which are important in depicting the effect produced by those biases. This study tried to construct a prediction model for physicians to figure out items that may demand additional consideration for interpretation or subsamples who tend to produce unexpected performance in CARS1.

## Material and methods

### Participants

This study was conducted with the approval of the Research and Ethics Committee of the Shenzhen Children’s Hospital. Children were recruited from the ASD Referral Project of the Disabled Persons Federation in Shenzhen, China. Children diagnosis as ASD or received suspected diagnosis as ASD were appointed to the Rehabilitation Department of Shenzhen Children’s Hospital for a multidisciplinary assessment through this project. The referred child can access necessary intervention once they achieved a definitive diagnosis of ASD based on the conclusive evidence. CARS1 is involved in the multidisciplinary assessment toolkit. A multidisciplinary team is invited to confirm the diagnosis of ASD. The members included a psychiatrist with ADOS-2 license and two senior neurologists.

Prior to administration, all the subjects and/or their legal guardians(s) had signed necessary consents. Subjects were included if they were older than 2 years of age and met the diagnostic criteria of the DSM-5-TR (version 2022), and the clinical presentation consisted of three manifestations of social disorders as well as any two manifestations of stereotyped repetitive behaviors, as follows:

### Socialization disorders

1. Social–emotional interaction disorders
2. Physical motor behavioral (nonverbal communication) social disorders
3. Social relationship development disorder (development, formation, understanding)

### Stereotypical repetitive behavior

1. Repetition of stereotyped motor movements, object manipulation, or verbal expressions
2. Development of repetitive, routine, and patterned stereotyped verbal or nonverbal behaviors
3. Extremely limited, fixed interests, or attention spans
4. Abnormal responses (extreme sensitivity or the opposite) to sensory input, both normal and abnormal (environmental)

For specific diagnostic criteria, refer to C.E. Rice’s suggested judgment criteria for each entry (19).

Patients with other unrelated conditions were excluded, including peripheral nerve injury, myelitis, spinal embolism syndrome, seizures, and fractures.

### Measure

CARS1 was constructed to collect information from major caregivers’ interviews, direct or indirect observations, and structural interviews. A total of 15 items are involved in CARS1, including relation to people, imitation, emotional response, body use, object use, adaptation to change, visual response, listening response, sensory response, emotion, verbal communication, gesture, activity status, intellectual response, and overall impressions. A four-point rating scale is utilized to quantify the symptoms severity, where one point refers to normal behavior and four points refers to inappropriate behaviors that are different from normal developed children. The total score is the sum of all items, and the higher scores refers to more severe autistic symptoms. CARS1 was delivered by trained/licensed clinicians or researchers with appropriate training for the necessary interviews techniques with parents and caregivers and judgement criteria.

### Data analysis

#### Rasch model

The Rasch model is widely utilized to test the psychometric properties of commonly used assessment tools including the Test of Infant Motor Development, Motor Proficiency 2nd Edition, Peabody Developmental Motor Scale, etc. (20–24). Our study chose the Partial Credit Model (PCM) to examine the construct validity of the CARS1.

Our study utilized the listened WINSTEPS software^1^ and R for all necessary analysis. Here, we hypothesize that, with more severe autistic symptoms, children with ASD may perform more in CARS1 items. That means children with more severe autistic symptoms obtain more scores than those who are less autistic. Although the CARS1 adopted the same rating scale for all items, the relative difficulties of the steps differed from item to item.

#### Category function

The CARS1 assigned 1 to 4 points to each item, and the points on the scale continuum where there is a fifty percents probability of scoring either of two adjacent points (e.g., 50% to get 1 point or 2 points) is considered as one threshold. Therefore, a 4-point rating scale contains three thresholds, and these locations/thresholds should be distributed in order on the scale continuum. This implies that individuals with more severe symptoms should be assigned higher scores. In addition, the gap between adjacent thresholds should cover a reasonable range on the continuum so that each item scale can distinguish individuals with different symptom levels. An ideal category structure should assign a reasonable number of participants to appropriate locations on the scale continuum (25–27). Therefore, the following criteria are recommended for assessing the category function: First, each option should receive at least ten responses. Second, the difficulty index of the category thresholds should increase monotonically. Third, the threshold interval should range from 1.4–5. Items that show disorder category functions are recommended to be rescored by collapsing adjacent categories, and a reanalysis is needed to check whether a better model is achieved.

#### Item fitness

The item and person scores were calculated based on nature logarithm. The prior hypothesis was tested by calculating the overall item and person fitness to the Rasch Model. To eliminate the erratic effects caused by unexpected responses, our study utilized infit mean square and standardized Z (Zstd) by assigning weights to the calculated residual. As previous studies suggested, the infit mean square and Zstd should fall within 0.75 to 1.33 and −2 to 2 respectively (28–30). A reasonable differential efficacy is established with a separation index over 2.0, and reliability is supported with an index beyond 0.8 (28–30).

#### Unidimensionality

The special decided principal component analysis was adopted to study the distribution of the differences between true and predicted performance. The unidimensional structure is validated if over 40% of the variance of the residual can be explained by the measurement dimension, and the distribution of the residuals explained by the extra dimension should follow random characteristics (eigenvalue less than 2.0) (29–31).

#### Differential item functioning

To investigate the consistent impact produced by the demographic variants, we try to establish a model named Generalised Partial Credit Model (GPCM) to simulate different scenarios under different parameter combos by using GPCMlasso R package. A lasso penalty was used to obtain the optimal loss function. In GPCM, *λ* denotes the influence of the covariances (e.g., age group, gender, symptom level in this article) on individual response probability, the λ magnitude refers to the variances impact which means the uniform DIF is denied once the coefficient equals to zero. Our study mainly focus on the following variants including gender, age group, and symptom level.

Therefore, the GPCMlasso model can be expressed as follows:


$$\log(\frac{P(Y_{\mathrm{pi}}=r)}{P(Y_{\mathrm{pi}}=r-1)})=\beta_{i}[\theta_{p}+x_{p}^{T}-\delta_{\mathrm{ir}}-(\begin{array}{l} \gamma_{i1}*\text{Gender}+\gamma_{i2}*\\ \text{AgeGroup}+\gamma_{i3}*\\ \text{Symptomlevel} \end{array})]$$

In this equation, $\gamma_{\text{in}}(n=1,2,3)$ denotes the impact of demographic variants on item i . To make amendments to the original DIF calculated by Welch’s t-test, a assumptions test used for unequal samples, the GPCMlasso model can test multiple covariates simultaneously and eliminate the potential multicollinearity that may exist among these variables. In this study, the Bayesian information criterion was adopted to screen for the optimal parameter, λ.

We conduct an extra t-test and an ANONA to compare the difference in CARS1 total scores between gender and among individuals from different age groups.

#### Sample consideration

To obtain 99% confidence that the item calibration (item difficulty measure) is within ±1/2 logit of its robust value and avoid type one errors, a sample between 250 and 500 is recommended (32–34).

## Results

### Demographic data

Our study managed to recruit 3,348 children and adolescents to contribute their performances to our study. Table 1 shows that this sample aged around 45.15 months old (45.15 ± 23.75 months). More boys joint in our study, that means the gender ratio was 2673/675 (male/female). To eliminate the educational impact, we try to collect a sample with balance in terms of educational background according to the Chinese Education System (kindergarten: three-six years old, primary school: six-twelve years, junior high school: twelve-fifteen years). However, we ultimately obtained a sample of 1,416 children fostered at home, 1506 children from local kindergarten, 406 primary students, and 20 children and adolescents from junior high school. In terms of symptom severity, we managed to obtain a sample with balance in the CARS severity classification, as shown in Table 1.

**Table 1:** Participant demographic data.

Variables	Mean (SD)/Count
Sample	3,348
Gender	
Male	2,673
Female	675
Age (months)	
Overall	45.15(23.75)
0–35/infant	1,416
36–71/kindergarten	1,506
72–143/primary	406
144–180/junior high	20
ASD symptom (CARS classification)	
Symptom level	31.69(5.38)
Non autism	1,114
Mild to moderate autism	1,635
Severe autism	599
tesing_timing (sec)	209(108)

S.D., standard deviation, CARS, children autism rating scale.

### Person and item mapping and fit statistics

**Figure 1:**
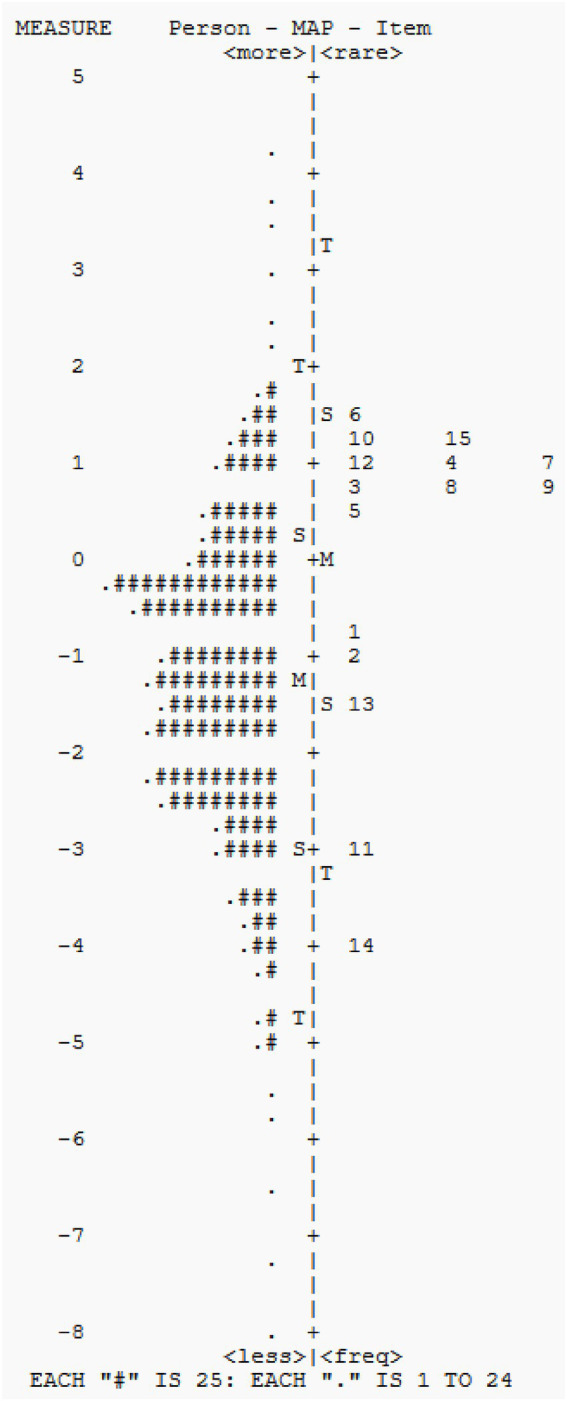
Person item map of the items in the CARS.

Figure 1 presents an overall item difficulty distribution. As shown in Figure 1, item 6 (“Adaptation to environment change”) was the most difficult. This means that maladaptation to environmental change may be a behavior that could only be observed in those with the most severe autistic symptoms. Item 14 (“Level and consistency of intellectual response”) was the easiest one which means that underdeveloped intellectual behavior may be the most seen symptom in this sample. As the mean item difficulty was set at 0 logit, Figure 1 shows that most of this sample may present with mild to moderate symptoms. In addition, CARS may not depict children with extremely mild or severe ASD symptoms. Figure 1 shows that some “.” and “#” are scattered outside the rating range, which means that no items can describe these children’s symptoms in detail.

Table 2 shows that we managed to simulate the response pattern of this sample by using Rasch Model. The person reliability and separation index shows that CARS can efficiently collect the inter-person symptomatic variants to present the autistic severity rather than other irrel.

**Table 2:** Fit statistics summary of the CARS.

Fit statistics		Total score	Count	Measure	Infit		Outfit		Real separation	Real reliability
					MNSQ	ZSTD	MNSQ	ZSTD		
Person	Mean	31.7	15	−1.33	0.97	−0.11	1.08	0.04	2.57	0.87
	S.D.	0.1	0	0.03	0.01	0.02	0.01	0.02		
Item	Mean	7074.7	3,348	0	1	−1.55	1.08	−1.32	39.52	1
	S.D.	322.1	0	0.43	0.09	2.15	0.14	2.16		

S.D., standard deviation.

Table 2 shows that no significant deviation is detected between the predicted and actual behavior, that means the response patterns of children with ASD can be well-simulated by probabilistic model. The inter-person differences measured by reliability and separation showed that CARS can well distinguish people with different symptoms or autistic severity. That means CARS are depicting inter-person symptomatic variations rather than other irrelevant symptoms.

A value of 0.87 means that 87% of the individual variations captured by the CARS are caused by inter-person differences, and 13% are due to random error. The item reliability and separation index showed that our sample size was large enough to determine the difficulty order of items on a hypothesis rating continuum.

### Evaluation of item fitness

Our analysis detected six items that displayed misfitting (over or unfitting) to the Rasch Model (Table 3). This means that individuals tend to deviate from the expected performance on these items, according to the Rasch Model.

**Table 3:** Fit statistics for unfitting (overfitting and misfitting) items.

Item	Content	Total score	Total count	Measure	Model S.E.	Infit		Outfit	
						MNSQ	ZSTD	MNSQ	ZSTD
1	Relating to people*	8,086	3,348	−0.81	0.04	**0.62**	**−9.9**	0.6	−9.9
2	Imitation	7,691	3,348	−0.91	0.03	0.88	**−4.73**	0.86	−5.33
3	Emotional response	6,923	3,348	0.77	0.04	0.78	**−9.12**	0.74	−9.09
4	Body use	6,377	3,348	1.08	0.04	1.03	1.05	1.05	1.67
5	Object use*	6,711	3,348	0.56	0.04	0.75	**−9.9**	0.71	−9.9
6	Adaptation to environment change	6,402	3,348	1.53	0.04	1.25	**8.31**	1.28	7.93
7	Visual response	7,249	3,348	1.02	0.03	0.83	**−7.98**	0.85	−6.46
8	Listening response*	7,669	3,348	0.81	0.03	**0.67**	**−9.9**	0.66	−9.9
9	Taste, smell, touch response and use	5,117	3,348	0.87	0.04	1.08	**3.27**	1.26	5.84
10	Fear or nervousness	5,051	3,348	1.26	0.04	1.23	**9.79**	1.66	9.9
11	Verbal communication	10,140	3,348	−3	0.04	1.1	**3.55**	1.1	3.18
12	Nonverbal communication	7,154	3,348	0.94	0.03	0.84	**−7.58**	0.82	−7.72
13	Activity level*	6,747	3,348	−1.46	0.04	**1.46**	**9.9**	1.59	9.9
14	Level and consistency of intellectual response*	8,194	3,348	−3.89	0.04	**1.82**	**9.9**	2.54	9.9
15	General impression*	6,609	3,348	1.25	0.04	**0.6**	**−9.9**	0.55	−9.9

*Unfitting or over-fitting items. Numbers in Bold indicate the numbers that violate the recommended range, the recommended range is 0.75-1.33.

### Category function

The category function analysis revealed that options received 4 points in all items except items 1, 2, and 11, which were not proportionally endorsed (less than 10 responses). This can be explained by the sample characteristics, as most of the sample displayed mild-to-moderate symptoms. Table 4 shows that all items displayed an ordered threshold measure. The magnitude of the interval between adjacent thresholds for some items violated the recommended ranges. This may be due to insufficient response in certain categories (e.g., category 4).

**Table 4:** Items with disorder category fucntion in CARS.

Items	Content	Value at threshold between categories^1^				
		Threshold 1 (Cat.1–2)	Threshold 2 (Cat.2–3)	Threshold 3 (Cat.3–4)	Interval Thr.1–2	Interval Thr.2–3
1	Relating to people	−4.86	−0.17	5.03	4.69	5.2*
2	Imitation	−3.87	0.7	3.17	4.57	2.47
3	Emotional response	−5.01	0.06	4.96	5.07*	4.9
4	Body use	−4.36	0.54	3.83	4.9	3.29
5	Object use	−4.29	0.51	3.78	4.8	3.27
6	Adaptation to environment change	−4.96	0.17	4.79	5.13*	4.62
7	Visual response	−4.65	−1.29	5.94	3.36	7.23*
8	Listening response	−4.47	−1.8	6.28	2.67	8.08*
9	Taste, smell, touch response and use	−2.12	2.12	-	4.24	0
10	Fear or nervousness	−2.49	2.49	-	4.98	0
11	Verbal communication	−2.71	−1	3.71	1.71	4.71
12	Nonverbal communication	−4.52	−1.05	5.58	3.47	6.63*
13	Activity level	−2.82	2.82	-	5.64*	0
14	Level and consistency of intellectual response	−2.8	2.8	-	5.6*	0
15	General impression	−4.71	−0.21	4.92	4.5	5.13*

^1^Cat: Category/option, Thr: Threshold, *abnormal interval.

### Assessment of unidimensionality

The principal component analysis of the residuals revealed that the variance explained by the measure was 54.7%, which is slightly higher than the value reported in a previous study (51%) (16). The variance explained by the 1st contrast was 6.9%, and the variance ratio of measures to the 1st contrast was larger than 3:1 (18.08/2.27).

The eigenvalue in 1st contrast was higher than 2. To determine which items contributed to the 1st contrast, 0.4 is a arbitrarily setting value as the cut-off value to determine a meaningful factor loading (Table 5). In addition, since the eigenvalue in 2nd contrast was 1.8, which is close to 2. We decided to view the 1st and 2nd contrast at the same time.

**Table 5:** Standardized residual loadings for item on 1st and 2nd contrast.

Item	Content	Loading	Measure	Infit MNSQ	Outfit MNSQ
1st					
1	Relating to people	0.45	−0.81	0.62	0.6
2	Imitation	0.59	−0.91	0.88	0.86
8	Listening response	0.54	0.81	0.67	0.66
11	Verbal communication	0.52	−3	1.1	1.1
12	Nonverbal communication	0.58	0.94	0.84	0.82
2nd					
1	Relating to people	0.39	−0.81	0.62	0.6
3	Emotional response	0.48	0.77	0.78	0.74
4	Body use	0.06	1.08	1.03	1.05
5	Object use	0.13	0.56	0.75	0.71
7	Visual response	0.57	1.02	0.83	0.85
8	Listening response	0.21	0.81	0.67	0.66
15	General impression	0.66	1.25	0.6	0.55

### Differential items functioning

DIF analysis was conducted using rescored data. Categories were collapsed to eliminate options with insufficient response. According to the BIC methods, our results showed that all items in the CARS displayed DIF differently by sex, age group, and symptom classification (Table 6). This implies that children and adolescents with different variables may perceive and interpret the meanings of the items differently. In the GPCMlasso equation, each group variable is replaced by the corresponding *λ*, and the predominant variables are set as the reference.

**Table 6:** The results of DIF analysis based on lasso coefficients in the GPCMlasso model for variables in the CARS.

Item	Content	Gender	Age Group			Symptom	
			Kindergarten	Primary	Junior high	Non-autism	Severe autism
1	Relating to people	0.096	0.554	0.454	0.127	−1.44	0.429
2	Imitation	0.05	1.325	1.303	0.235	−1.178	−0.058
3	Emotional response	0.1	−0.278	−0.475	−0.186	−1.024	0.023
4	Body use	0	−0.169	−0.346	−0.283	−0.906	−0.943
5	Object use	−0.241	0.378	0.012	0	−0.38	−0.33
6	Adaptation to environment change	0	−1.384	0	−0.115	3.848	−0.671
7	Visual response	0.117	−0.205	−0.202	0.054	−1.176	0
8	Listening response	0.087	0.764	0.687	0.196	−1.508	0.306
9	Taste, smell, touch response and use	0.149	−0.303	−0.717	−0.456	0	−2.37
10	Fear or nervousness	1.116	−2.103	−2.376	−2.358	4.946	−5.553
11	Verbal communication	0.092	2.091	1.822	0.417	−1.128	−0.056
12	Nonverbal communication	0.142	1.084	0.911	0.216	−1.286	0.065
13	Activity level	−2.727	−2.218	−1.477	0	3.124	−1.049
14	Level and consistency of intellectual response	−0.702	−1.687	0	0	0.743	3.12
15	General impression	0	0.123	−0.097	0.175	−1.341	−0.068

To simplify the original formulation, the GPCMlasso Model can be written as follows:


$$\log(\frac{P(Y_{\mathrm{pi}}=r)}{P(Y_{\mathrm{pi}}=r-1)})=[\theta_{p}-(\begin{array}{l} \beta_{i}+\gamma_{i1}*\text{Gender}+\\ \gamma_{i2}*\text{AgeGroup}+\\ \gamma_{i3}*\text{Symptomlevel} \end{array})]$$

For illustration, this study recruited a sample that predominantly consisted of men. Then, Gender/male was equal to zero, and Gender/female was equal to 1. Hence, the difficulty can be $\beta_{i}$ for males and $\beta_{i}+\gamma_{i1}*\text{Gender}/\text{female}\;$ for females. The Lasso coefficient $\gamma_{i}$ represents the deviation of the subsamples in item difficulty from the baseline difficulty $\beta_{i}$ . If $\;\gamma_{i}\;$ is equal to zero, the corresponding group variables function equally to the baseline variables.

Our study adopted a t-test and an ANOVA analysis to depict the difference among subgroups. Table 7 shows that no significant difference is found between gender. That means those testing bias brough by gender did not affect the CARS1 total score. ANOVA analysis showed that older children are achieving lower scores especially when they enter primary school.

**Table 7:** The CAR1 scores comparison in DIF analysis regarding gender and age group.

Components	Boys/N^1^	Girls/N	*p* ^2^	infant	kindergarten	primary school	junior high school	*p*	Bonferroni t-test^3^
	675	2,673		1,416	1,506	406	20		
CARS1 sum	31.46 ± 5.28	31.74 ± 5.36	0.65	32.13 ± 4.92	31.85 ± 5.11	29.55 ± 6.89	30.8 ± 5.69	0.00	1,2 > 3

^1^*N*, sample size; ^2^*p*, *p* value; ^3^ 1, infant, 2, kindergarten, 3, primary school, 4, junior high school.

## Discussion

In this study, we utilized machine learning methods in creating a probabilistic model to simulate personal performance in CARS, so that we can depict the psychometric properties of CARS in different perspectives. CARS was widely used in clinical assessment in population diagnosis as ASD with different demographic traits (e.g., age, gender, symptom profiles) in different clinical settings and research scenarios. The psychometric properties were already well illustrated based on classical testing theory in pervious works done by other researchers. Hence, robust results can be expected at a scale level to depict the symptom status of children with ASD. However, a relatively new probabilistic model named Rasch Model can provide more detailed psychometric evidence at item level, hence we conduct this to emphasize some limitations regarding potential measurement bias mentioned previous work. In brief, we found that PCM is a reasonable model to simulate the responding pattern to CARS of children with ASD. In line with previous work, at a scale level, CARS can explain 54.7% (over40%) of the measurement variance. Our work confirmed that CARS are depicting the autistic symptoms based on a unidimensional base. Our study also found some limitations regarding the item category that the current rating scale is so rigid that some minor behavior difference would be missed. That means items may provide inaccurate information originating from unreasonable threshold intervals. We also discover some critical demographic variables with potential threads to produce obvious assessment bias which may contribute to unstable psychometric quality reported in previous findings.

### Measurement properties of the CARS items

The overall response pattern of individuals with ASD showed reasonable fitness for the PCM. This means that individuals with more severe ASD symptoms had higher CARS scores. However, some items were not adequately endorsed by the experts. For instance, item 3 (“Emotional response”) did not receive enough responses on a 4 point (5/3348), and only five people rated this item with a score of 4. This can be partially explained by the sample characteristics; this study included 599 individuals with severe ASD symptoms, but this subsample may not have perceived the corresponding concerns in emotional response. Item statistics showed that some individuals tended to deviate from the expected pattern based on the Rasch model. This can be attributed to the unreasonable threshold intervals of the CARS. For example, the interval between thresholds 2 and 3 for item 1 (“relating to people”) is wider than the recommended range (5.2 > 5). This means that the ability interval between Threshold 2 (the interaction between Points 2 and 3) and Threshold 3 (the interaction between Points 3 and 4) accommodates too many individuals with a wide range of abilities in this continuum. Those who rated their symptoms as 2 or 4 tended to fall within the measurement interval defined by three points. These unreasonable intervals may produce unnecessary fluctuations or false information in clinical practice.

### Item difficulty hierarchy and unidimensionality

The item-person map shows that certain items tend to cluster on the upper part of the scale; hence, individuals with moderate to severe ASD symptoms can be depicted in more detail. However, patients with mild symptoms may not benefit from CARS. In this study, the participants were mainly clustered around the 0 logit unit. The difference in distribution between the participants and items indicates that items may not be adequately endorsed by this sample, as observed in the present study.

As a previous study reported, CARS can explain over 50% of the measurement variance. Although the analysis revealed one meaningful extra contrast within the CARS, no specific item showed a significant contribution. Hence, we suggest that the CARS be constructed purely based on a theoretical structure. The remaining variance that cannot be explained by the measurement may be caused by the random noise.

### Differential item functioning

To date, this study is the first to focus on the DIF analysis of the CARS1. In addition, this study is the first to use machine learning methods for the DIF analysis. According to the BIC method, our results revealed that 15 items in the CARS1 displayed different DIF in different groupings.

Our results revealed that only item 4 (“body use”), item 6 (“adaptation to environment change”), and item 15 (“general impression”) did not display gender DIF. This implies that boys and girls perceive and interpret these items equally. The other results indicate that age groupings and symptom classifications can alter the probability of item endorsement on the CARS. At the item level, we found that item 1 (“relating to people”), item 2 (“imitation”), item 5 (“object use”), item 8 (“listening response”), item 11 and 12 (“verbal and non-verbal communication”) were more commonly noticed as children matured. To our surprise, individuals with fewer autistic symptoms should be endorsed with less probability to rate higher scores in the CARS, but we found that this subsample (non-autism in this study) tended to rate high scores in 10 of the 15 items in the CARS. One possible explanation is that we found the *λ* index for the non-autism subsample to be −0.272 and −1.273 for severe autism in the CARS scale. This means that this non-autistic subsample may score low points that fail to meet the criteria, but their behavior may impress clinicians and caregivers that they may be children with ASD.

Our study utilized machine learning methods to solve the statistical problem regarding multicollinearity and covariates calibration which is addressed in previous DIF analysis research. In summary, our simulation model reveal that children with ASD perform differently depending on educational background or age groups, gender, and symptom severity.

### Implications for clinical practice

Our findings confirmed the hypothesis that children with more severe autistic symptoms can display more problematic behaviors, hence CARS can be a practical tool to quantify the symptom severity in children with ASD. Our study also provides some useful clues when a clinical decision is needed using CARS scores merely less or larger than cutoff scores. At that time, age groups or educational resources, gender and symptom severity can be helpful to make critical decisions.

### Study limitation

For statistical considerations, we cannot deny some DIF may show up in non-uniform form, but it is relatively complicated to simulate such irregular scenarios using penalized likelihood functions. We believed that behavioral observation involves more than simply demographic factors, but our study design had limited the possibility to involve more possible and meaningful neurodevelopmental comorbities to calibrate CARS1. Moreover, we only recruit 20 children from junior high school, and this may jeopardize the model performance in explaining the behaviors of children with older age or higher educational background.

## Conclusion

In line with previous reporting, CARS is a convenient tool in depicting autistic symptoms in children with ASD. However, we also found some significant limitations that may jeopardize the psychometric properties which are confirmed in previous findings. Some items may generate inaccurate evidence due to inappropriate scoring rules or standards. On the other hand, children with ASD are performing differently in CARS depending on various demographic traits including age or educational grouping, gender and autistic severity. This finding may serve as hints in making clinical decision upon scores merely smaller or larger than cutoff scores.

## Data Availability

The raw data supporting the conclusions of this article will be made available by the authors, without undue reservation.

## Footnotes

^1^ http://www.winsteps.com
